# Assessment of left ventricular systolic function after VSD transcatheter device closure using speckle tracking echocardiography

**DOI:** 10.1186/s43044-019-0001-7

**Published:** 2019-08-05

**Authors:** Yasmin Abdelrazek Ali, Maryam Araby Hassan, Azza Abdallah EL Fiky

**Affiliations:** Ain Shams University, Cairo, 11528 Egypt

**Keywords:** Ventricular septal defect, Speckle tracking, 2D strain, Transcatheter VSD closure

## Abstract

**Background:**

This is a case-control study conducted on 30 children, 15 with VSD who performed VSD transcatheter device closure (group A) and 15 controls of matching age and gender (group B), in the period between September 2015 and February 2018. We aimed to assess the global left ventricular (LV) systolic function by 2D speckle tracking before and after ventricular septal defect (VSD) transcatheter closure, in comparison to normal controls. All patients were subjected to full history taking; general and cardiac examination; ECG; CXR; full transthoracic echocardiographic examination, including VSD number, size, and site; LV dimensions and volumes; estimated pulmonary artery pressure; right ventricular size and function; left ventricular circumferential; and radial strain imaging by 2D speckle tracking. Patients who had ventricular septal defect closed were reassessed by transthoracic echocardiography after 3 months.

**Results:**

The study included 15 children with VSD: 3 males and 12 females; their age ranged from 2 to 13 years; all had subaortic VSD except for 1 who had apical muscular VSD: VSD size ranged from 3 to 8 mm; PFM coil was used to close defect in all patients except for 2 patients who had an Amplatzer duct occlude I (ADOI) device, and 1 patient needed an additional vascular plug after significant hemolysis. Pre-procedurally, group A had a significantly higher LVEDD, LVESD, and LVEDV than group B. Mean circumferential strain was significantly higher (more negative) in group A than that in group B either pre- or post-procedure. Post-procedurally, there was a significant decrease in circumferential strain (less negative) and a significant increase in radial strain (more positive).

**Conclusion:**

Following transcatheter VSD closure, there is a significant decrease in LV circumferential strain and a significant increase in LV radial strain, which conclude a decrease in LV volume overload with the improvement of its contractility.

## Background

Ventricular septal defect (VSD) is the most common congenital heart disease accounting for 40% of all congenital heart diseases. An isolated VSD accounts for more than 20% of all congenital heart diseases [1]. It is classified according to its relation to septum as inlet, trabecular, outlet, and membranous septum [2]. Another classification is based on VSD location on the right surface of the interventricular septum as single or multiple, infundibular, perimembranous, inlet, muscular, and Gerbode defect [3].

Management in the infant and child depends on symptoms; children with small asymptomatic defects need no medical management and are unlikely to need any intervention. First-line treatment for moderate or large defects affecting feeding and growth is with medical treatment for heart failure and high-energy feeds to improve calorie intake. Any patient needing significant medical management should be referred for surgical assessment [4].

For a long time, the only way to close VSDs was open-heart surgery. This is a major procedure that necessitates a thoracotomy, heart-lung bypass, blood transfusion in some cases, permanent scar and potential risks of complete heart block, residual shunt, early and late arrhythmias, and post-pericardiotomy syndrome [5], yet surgical closure has been performed with low perioperative mortality and a high closure rate [6]. Nowadays, successful transcatheter device closure of trabecular (muscular) and perimembranous VSDs has been performed. Trabecular VSDs have proven to be more amenable to this technique with excellent closure rates and a low procedural mortality [7–10].

The most widely used echocardiographic parameter to quantify left ventricle (LV) systolic function has been LV ejection fraction (LVEF). While LVEF is a strong predictor of mortality and is used to select patients for device implantation [11], surgical procedures [12], and pharmacological treatments [13], it is extremely load-dependent, its measurement with echocardiography depends critically on operator expertise, and it is affected by significant intra-observer and inter-observer variability [14].

Newer two-dimensional speckle tracking imaging (2D STI) allows for quantitative investigation of global and regional myocardial wall motion and deformation [15, 16], and its practical application has also been reported in pediatric cases [17, 18].

After surgical VSD closure, 2D STI detected significant regional depression at the mid-ventricular level according to the peak circumferential strain variables, with no significant regional depression observed according to the peak systolic radial strain variables [18]. Therefore, we used 2D STI to assess left ventricular systolic function pre-transcatheter and 3 months post-transcatheter VSD closure, to assess its impact on LV circumferential and radial myocardial deformation and hence LV systolic function.

## Methods

This study was conducted on 30 children, 15 patients with VSD who were referred to perform VSD transcatheter device closure and 15 control children (with structurally and functionally normal heart and of matching age and gender), in the period between September 2015 and February 2018.

Patients with contraindication to transcatheter closure, i.e., large size VSD, Eisenmenger’s syndrome, other associated congenital heart diseases, rheumatic heart diseases, associated cardiomyopathy, irregular rhythm, or baseline LV dysfunction before VSD closure, were excluded from the study.

After the approval of the Ain Shams University ethical committee, written informed consent was obtained from all the patients’ parents. All patients were subjected to full history taking including age, gender, history of parents’ consanguineous marriage, drug intake during pregnancy or exposure to irradiation, recurrent chest infection, delayed growth milestones, history of dyspnea, previous catheterization or surgery, time of VSD transcatheter closure, and cardiac catheterization details (size and number of VSDs by angiography, device used for closure and its size, final result, any complications, history of any degree of post-procedural heart block, and how it was managed).

Patients were examined for developmental milestones, signs of other congenital anomalies, or syndromic features, with an assessment of body weight (kg), height (cm), and surface area (calculated using Haycock formula). Cardiac examination for the assessment of precordial bulge, scars of previous surgery, dextrocardia, left parasternal thrill, hyperdynamic apex, auscultation for heart sounds, additional heart sounds (e.g., accentuated S2, S4, gallop rhythm), systolic murmurs, and signs of cardiac pump failure (pulmonary or systemic venous congestion) was done.

Standard 12-lead surface electrocardiography (ECG) before and 3 months after the procedure was done to assess the following: rate, axis, arrhythmias (especially post-procedure to rule out any degree of heart block), chest X-ray (CXR), and routine laboratory investigations including complete blood count, liver and renal function tests, and bleeding profile.

All echocardiographic measurements were obtained at baseline and more than 3 months after the procedure using a Philips IE33 echocardiography machine, with S5-1 transducer. Pediatric patients who could not lie still were sedated using chloral hydrate in a dose of 50 mg/kg. The complete diagnostic transthoracic examination was done by 2D echo, Doppler, and color flow mapping, applying the sequential analysis technique from all available windows, identifying the size and site of VSD, number of VSDs if multiple, and direction and pattern of flow across.

**Fig. 1:**
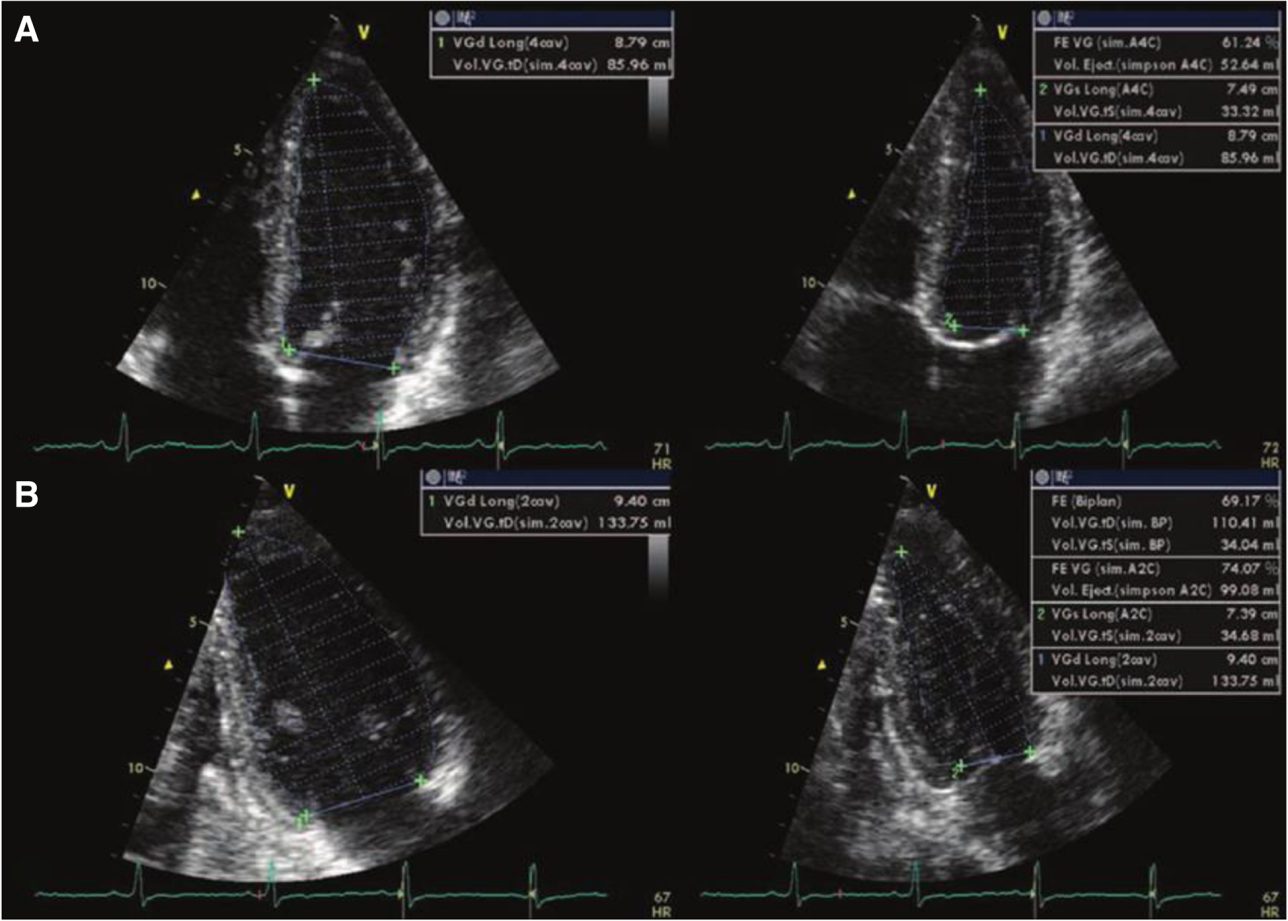
LV end-diastolic and end-systolic volume by Simpson 4-chamber (**a**) and LV end-diastolic and end-systolic volume by Biplane Simpson 2-chamber method (**b**). LV (left ventricle)

LV assessment by m-mode in PSAX view at the level of MV chordae was done to assess LVEDD (left ventricle end-diastolic dimension), LVESD (left ventricle end-systolic dimension), wall thickness, and EF (ejection fraction) and by the modified Simpson method from apical 4-chamber and 2-chamber views to assess LVEDV (left ventricle end-diastolic volume), LVESV (left ventricle end-systolic volume), and EF (Fig. 1).

Circumferential and radial strain imaging by 2D speckle tracking echocardiography was done as follows: high-quality ECG-gated images from the parasternal short-axis (basal, mid, apical) views were obtained. All images were stored in cine-loop format from three consecutive beats, views were optimized through changing the transducer scan width to achieve a frame rate of at least 40 Hz, and data were transferred to a workstation for further offline analysis to assess the peak circumferential and radial 2D strain.

**Fig. 2:**
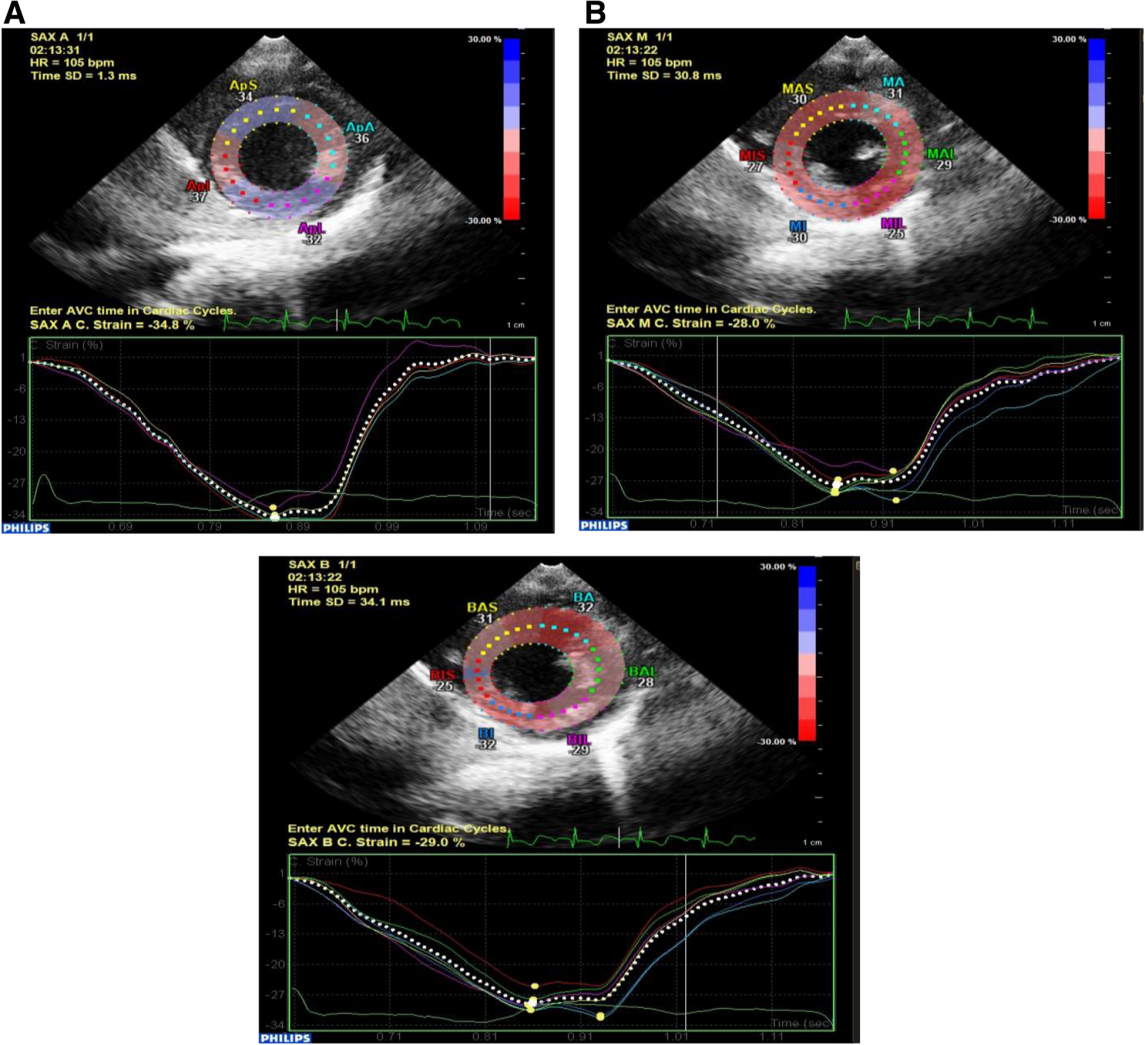
Measurement of circumferential strain in the left ventricular short-axis views at apical (**a**), mitral valve (**b**), and basal (**c**) level with the region of interest at each level

Speckle tracking analysis for the LV was performed and consisted of marking the endocardium, defining the width of the region of interest, reflecting the distance from the endocardium to the pericardium, and running the automatic analytic algorithm. Within the defined region of interest, the software performed motion analysis of natural acoustic markers (speckles). The circumferential and radial peak systolic strain was calculated for each segment (Fig. 2).

The operator who performed the follow-up TTE (transthoracic echocardiography) after 3 months was blinded to the pre-procedural TTE results and any clinical or catheterization data.

Normal mean values of circumferential strain (CS) varied from − 10.5 to − 27.0 (mean, − 22.06; 95% CI, − 21.5 to − 22.5). Radial strain (RS) normal mean values varied from 24.9 to 62.1 (mean, 45.4; 95% CI, 43.0 to 47.8 from the meta-analysis). Table 1 shows the suggested normal values for LV CS and RS in children [19].

**Table 1:** Reference mean values of left ventricle strain measures on Philips machine by vendor

Age distribution	Mean GCS (CI)	Mean GRS (CI)
0–1	NA	
2–9	− 22.4% (− 23.2, − 21.6)	NA
10–13	− 19.0% (− 19.6, − 18.4)	
14–21	− 18.0% (− 20.2, − 15.9)	NA
Overall	− 18.4% (− 19.7, − 17.1)	NA

Data presented as mean; *NA* not applicable (no studies in this age range), *GCS* global circumferential strain, *GRS* global radial strain

**Fig. 3:**
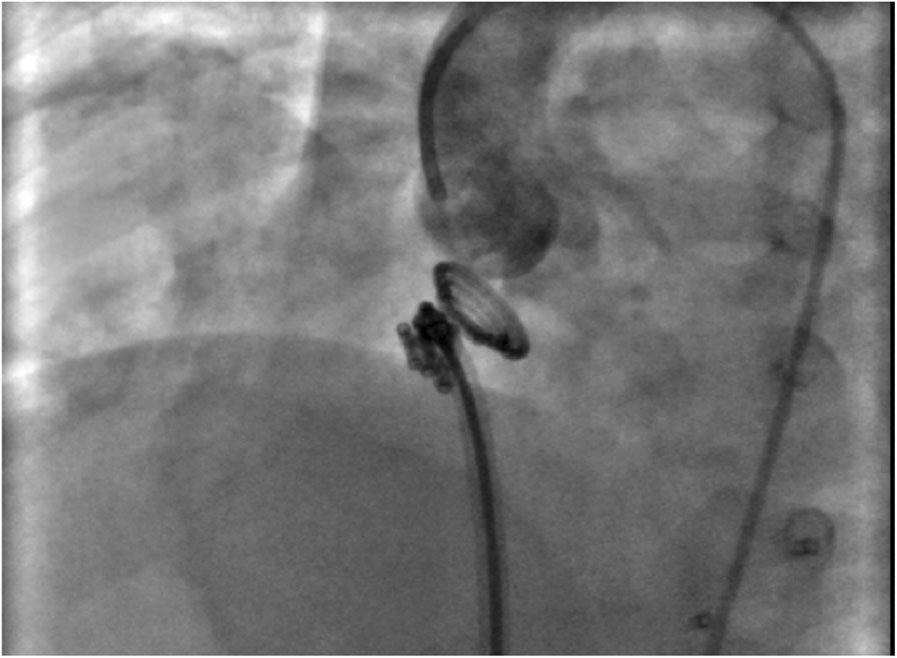
Aortography during VSD PFM coil closure before coil release

The transcatheter VSD device closure was done as follows: patients received general anesthesia on 100% oxygen under TEE (transesophageal echocardiography) guidance; full hemodynamic study was done including the assessment of pulmonary artery pressure, systemic pressure, LV and RV (right ventricle) saturation, assessed VSD size, site, number from LV angiography in LAO 60, and cranial 30 projection with proper selection of size of the device accordingly; the device size (waist diameter) was selected to be 1–2 mm larger than the largest measured diameter of the defect. After device positioning and before its release, proper positioning, residual flow, and any affection on aortic, mitral, or tricuspid valves flow were properly assessed by TEE and LV angiography (Fig. 3). The device was reassessed after its release by TEE and LV angiography again for proper positioning and residual flow. After complete recovery from anesthesia, patients were transferred to the pediatric care unit. Routine ECG to identify the rhythm and follow-up CXR were performed the following day to assure device position. Patients were kept on acetylsalicylic acid daily for at least 6 months.

### Statistical analysis

Data were coded and entered using the statistical package SPSS (Statistical Package for the Social Sciences) version 24. Variables were expressed as mean ± standard deviation (SD). Comparisons between quantitative variables of groups were done using the non-parametric Mann-Whitney test. For comparison of serial measurements within each patient, the non-parametric Wilcoxon signed-rank test was used. Correlations between quantitative variables were done using the Spearman correlation coefficient. *p* values less than 0.05 were considered as statistically significant.

## Results

The study was conducted on 15 patients: 3 males (20%) and 12 females (80%); their age ranged from 2 to 13 years (6.57 ± 3.62 years). Their weight ranged from 11 to 48 kg (22.89 ± 11.56), height ranged from 65 to 140 cm (114.8 ± 19.36), body mass index (BMI) ranged from 9.98 to 22.96 kg/m^2^ (16.23 ± 3.65), and body surface area (BSA) ranged from 0.52 to 1.57 m^2^ (0.79 ± 0.24) (Table 2).

**Table 2:** Demographic data of the study population

	Mean	SD	Minimum	Maximum
Age (years)	6.57	3.62	2	13
Weight (kg)	22.89	11.56	11	48
Height (cm)	114.8	19.36	65	140
BMI (kg/m^2^)	16.23	3.65	9.98	22.96
BSA (m^2^)	0.79	0.24	0.52	1.57

*SD* standard deviation, *BMI* body mass index, *BSA* body surface area

All patients were asymptomatic except for 8 patients who had a recurrent chest infection. On the examination, only seven patients had shifted hyperdynamic apex denoting dilated LV. All patients had a harsh pan-systolic murmur of VSD on auscultation, and five patients had a soft pan-systolic murmur of MR with maximum intensity over the apex radiating to the axilla. All patients’ laboratory investigations were within normal.

All patients had successful transcatheter closure of their VSDs as defined by Carminati et al. in 2007 (procedural success means device implantation in the appropriate position with no need for surgery, for example, due to significant residual shunt or significant valve regurgitation) [20].

All patients had normal sinus rhythm as confirmed by their ECGs pre-procedurally. Two patients had complicating sinus bradycardia after VSD closure, one of them had resting HR of 53 bpm with first-degree heart block (first-degree HB resolved 24 h after the procedure) and the other had a resting heart rate of 55 bpm. Both patients were asymptomatic and were followed up.

All VSDs were subaortic perimembranous except for 1 patient who had apical muscular VSD. Using transthoracic echocardiography, the VSD’s size from the left ventricular side ranged from 5 to 14 mm with a mean size of 10.8 mm and from the right side ranged from 3 to 8 mm with a mean size of 5.3 mm. All patients had one VSD except for 2 patients, who had 2 (subaortic perimembranous) VSDs. PFM coil was used for all patients except for 2 patients where the ADO I (Amplatzer duct occluder I) device was used (one of them had subaortic VSD with muscular extension, another one had apical muscular VSD). One patient needed an additional vascular plug (Table 3).

**Table 3:** Device types and sizes

Patient number	Device type	Device size
1	PFM coil	14 × 8 mm
2	PFM coil	10 × 6 mm
3	PFM coil	16 × 10 mm
4	PFM coil	14 × 8 mm
5	ADO I (duct occluder I)	8 × 6 mm
6	PFM coil	10 × 6 mm
7	PFM coil	8 × 6 mm
8	PFM coil	14 × 8 mm
9	PFM coil	14 × 8 mm
10	PFM coil	14 × 8 mm
11	PFM coil	14 × 8 mm
12	PFM coil	16 × 8 mm
13	PFM coil	12 × 6 mm
14	PFM coil	12 × 6 mm
15	ADO I (duct occluder I)	8 × 6 mm

Ten patients (66.7%) passed smoothly without complications while five patients (33.3%) had complications in the form of the following: (1) mild AR post-procedure, (2) trivial TR pre-procedure which became mild TR post-procedure, (3) asymptomatic sinus bradycardia post-procedure, (4) asymptomatic sinus bradycardia and first-degree heart block that resolved spontaneously, and (5) significant hemolysis and residual defect managed by an additional vascular plug.

There was minimal residual flow (1–2 mm by color Doppler) seen immediately after device implantation in 10 patients (66.6%) by TEE. After 3 months of follow-up, it decreased to 5 patients only (33.3%).

Echocardiographic parameters changed in group A (pre- and post-procedure) including the LVESD (24.6 ± 3.02 vs. 23 ± 2.75), LVEDD (38.87 ± 4.41 vs. 37.4 ± 4.69), LVESV (22.8 ± 5.78 vs. 20.73 ± 5.33), and LVEDV (62.27 ± 21.22 vs. 54.8 ± 18.8); all had an insignificant decline after device closure, with an insignificant increase in LVEF (63.73 ± 5.87 cm^2^ vs. 56 ± 5.39 cm^2^) before and after the procedure (Table 4).

**Table 4:** Changes in echocardiographic parameters after device closure in group A

Parameter		Mean ± SD	*p* value
LVESD (mm)	Pre	24.6 ± 3.02	0.177
	Post	23 ± 2.75	
LVEDD (mm)	Pre	38.87 ± 4.41	0.622
	Post	37.4 ± 4.69	
LVESV (ml)	Pre	22.8 ± 5.78	0.263
	Post	20.73 ± 5.33	
LVEDV (ml)	Pre	62.27 ± 21.22	0.384
	Post	54.8 ± 18.8	
LVEF (%)	Pre	63.73 ± 5.87	0.429
	Post	65 ± 5.39	

*LVESD* left ventricle end-systolic diameter, *LVEDD* left ventricle end-diastolic diameter, *LVESV* left ventricle end-systolic volume, *LVEDV* left ventricle end-diastolic volume, *LVEF* left ventricle ejection fraction

Comparison between group A and group B was done according to LVESD, LVEDD, LVESV, LVEDV, and LVEF. Pre-procedure LVESD, LVEDD, and LVEDV of group A are significantly higher than those of group B. No significant difference was noticed between the two groups according to other echocardiographic parameters (Table 5).

**Table 5:** Comparison between LV dimensions of group A and group B

Item		Group A			Group B				*p* value
	Mean	SD	Minimum	Maximum	Mean	SD	Minimum	Maximum	
Pre-LVESD	24.6	3.02	20	31	22.53	1.99	20	25	0.025*
Post-LVESD	23	2.75	17	29					0.387
Pre-LVEDD	38.87	4.41	31	49	36.07	2.46	31	41	0.005*
Post-LVEDD	37.4	4.69	32	45					0.234
Pre-LVEF	63.73	5.87	54	77	66.33	3.98	60	70	0.168
Post-LVEF	65	5.4	59	78					0.509
Pre-LVESV	22.8	5.78	16	37	18.8	3	15	23	0.711
Post-LVESV	20.73	5.33	15	32					0.208
Pre-LVEDV	62.27	21.22	30	117	48.07	9.54	30	69	0.002*
Post-LVEDV	54.8	18.8	33	90					0.263

*LVESD* left ventricle end-systolic diameter, *LVEDD* left ventricle end-diastolic diameter, *LVESV* left ventricle end-systolic volume, *LVEDV* left ventricle end-diastolic volume, *LVEF* left ventricle ejection fraction

*with statistically significant value

Mean circumferential strain (CS) of group A (pre- and post-procedure) was significantly higher than that of group B with *p* = 0.000056 and *p* = 0.019 respectively, whereas the mean radial strain (RS) of group A (pre- and post-procedure) did not have a significant difference from group B, *p* = 0.826 and *p* = 0.209 respectively (Table 6).

**Table 6:** Comparison between CS and RS for group A and group B

	Mean	SD	Minimum	Maximum	*p* value
Group A pre-CS	− 28.37	2.59	− 20.2	− 31.5	0.000056*
Group B CS	− 24.07	2.37	− 20	− 27	
Group A post-CS	− 26.59	3.13	− 19.3	− 32.9	0.019*
Group B CS	− 24.07	2.37	− 20	− 27	
Group A pre-RS	26.08	3.04	20.6	30.6	0.826
Group B RS	26.33	3.22	22	30	
Group A post-RS	27.72	2.68	21.6	31.8	0.209
Group B RS	26.33	3.22	22	30	

*CS* circumferential strain, *RS* radial strain

*with statistically significant value

There was a significant decrease in CS (less negative) and a significant increase in radial strain (more positive) pre- and post-procedure, where the mean pre-procedural CS was − 28.37 ± 2.59 and post-procedural CS was − 26.59 ± 3.13%, *p* = 0.015. The mean pre-procedural RS was 26.08 ± 3.04 and post-procedural RS was 27.72 ± 2.68%, *p* = 0.034 (Table 7).

**Table 7:** Periprocedural change in circumferential and radial strains

	Mean	SD	Minimum	Maximum	*p* value
Group A pre-CS	− 28.37	2.59	− 20.2	− 31.5	0.015*
Group A post-CS	− 26.59	3.13	− 19.3	− 32.9	
Group A pre-RS	26.08	3.04	20.6	30.6	0.034*
Group A post-RS	27.72	2.68	21.6	31.8	

*CS* circumferential strain, *RS* radial strain

*with statistically significant value

## Discussion

Although surgical option is the gold standard in the management of ventricular septal defect, percutaneous closure of VSD can achieve good results in closing perimembranous and muscular VSDs with advantages of reduced psychological impact, less pain, shorter hospital stays, no need for admission to an intensive care unit, faster time to normal activities, and less mortality [21, 22].

Our study included 30 individuals, with a mean age of 6.5 years. This is the same mean age of Pawelec-Wojtalik et al.’s [23] and Xunmin et al.’s [24] studied population. This is because percutaneous closure is usually done in patients with moderate-sized VSDs with normal pulmonary artery pressure and evidence of left ventricular overload which may possibly have developed over time.

In the current study, there was an insignificant decline in both LVEDD and LVESD; this differs from Pawelec-Wojtalik et al. [23], who reported that the left ventricular diameter decreased significantly after 1 year of VSD closure. Yang et al. [25] reported a decrease in LVEDD *Z* score from + 1.7 to + 0.7 (*p* < 0.001) after 2 years follow-up. This can be explained by the short follow-up duration (only 3 months) in our population which could not detect the reduction in LVEDD.

In the current study, there was no change in LVEF after transcatheter closure, and this result was concordant with Pawelec-Wojtalik et al. [23], who reported that the systolic function was not changed after VSD closure. This can be explained by the fact that patients who undergo VSD transcatheter closure mostly have moderate-sized restrictive defects and so the LVEF changes are subtle.

Speckle tracking echocardiography is a recently developed noninvasive ultrasound imaging technique for the quantitative evaluation of LV regional and global systolic function to detect subtle changes in the myocardial function [15, 26]*.* That is why we used this technique in this novel study to assess patients before and after transcatheter VSD closure.

We noticed that there was a significant increase in RS (26.08 vs. 27.72), which may conclude improvement of LV function after VSD transcatheter closure and a significant decrease in CS (− 28.37 vs. − 26.59) which may return to the abolishment of volume overload after VSD transcatheter closure.

Elsheikh et al. [27] studied strain values in patients with ASD before and after device closure. They concluded that volume overload significantly increases strain values of the right ventricle and these values return to normal after the closure of cardiac defect and abolishment of volume overload.

In the current study, the mean CS of patients before device closure was significantly higher than that of the control group. This is justified by volume overload caused by the VSD in all of our patients. The mean CS values decreased after VSD closure; however, they did not reach the control values. This can be due to short post-closure follow-up period (3 months only). We expect that longer follow-up is needed for these values to reach the control values.

In our study, there was minimal residual flow seen immediately after device implantation in 66.6% of patients by TEE. After 3 months of follow-up, it decreased to 33.3%. This may be explained by subsequent endothelialization. The residual flow was related to the presence of aneurysmal tissue. Haas et al. [28] reported trivial residual shunt immediately after percutaneous closure of perimembranous VSD with PFM coil in 51 out of 111 patients (50.0%), which decreased to 11% at 3 months, 5% at 6 months, 3% at 12 months, 1% at 24 months, and 0% at 36 months. The mechanism of closure in PFM coil involves filling up the defect with the device without real stenting [29].

Secondary valve dysfunction was present in 13% of patients in the current study. Mild aortic regurgitation is possibly related to the traction of the coil on the aortic valve after crossing of the defect and during delivery of the left ventricular side of the coil. Tricuspid valve affection was related to the close proximity of the defect to the atrioventricular valves. We concluded that the technical aspects during device implantation, disruption of chordae tendineae, and close proximity of the defect to the affected valves are possible contributing factors to valvular regurgitation.

Yang et al. [25] reported that the secondary impairments of the tricuspid and aortic valve seem common with a rate of 4.9%. The rate of permanent valvular regurgitation was 2.3% with tricuspid regurgitation in 1.7% and aortic regurgitation in 2.0%. Haas et al. [28] reported that 5% of patients developed mild tricuspid regurgitation and no one developed aortic regurgitation after percutaneous closure of perimembranous VSD using PFM coil.

In our patients, only one patient suffered from hemolytic anemia that required blood transfusion and re-intervention to close the residual shunt. She needed another vascular plug of 10 mm to completely occlude the residual VSD. Holzer et al. [29] and Rodriguez et al. [30] each had a similar case that suffered significant hemolysis and required re-intervention.

Haas et al. [28] reported the development of hemolysis in four patients out of 111 patients. In two patients, it resolved spontaneously. One patient required blood transfusion, and after implantation of the second device, the patient recovered immediately. One patient showed mild hemolysis even after 2 years of closure, and due to device displacement, surgical removal of the coil and VSD surgical closure were done.

Spence et al. [31] and Odemis et al. [32] reported that hemolysis is a rare complication that can cause significant sequelae. The rate of hemolysis following transcatheter VSD closure ranges from 0.7 to 15%.

We achieved successful closure in 100% of the cases. This may be due to the restrictive inclusion criteria for VSD transcatheter closure. This agrees with Rodriguez et al. [30], who concluded that percutaneous closure of perimembranous ventricular septal defect can be performed with a high success rate using PFM coil due to its special configuration.

## Conclusion

In the current study, there was a significant increase in RS and a significant decrease in CS after VSD transcatheter closure, which conclude the decrease in LV volume overload with the improvement of its contractility. To the best of our knowledge, this topic was never published before. We are aiming to continue our work by increasing the patients’ number and follow-up duration to confirm the results.

## Data Availability

The datasets used and analyzed during the current study are available from the corresponding author on reasonable request.

## Abbreviations

2D STI: 2D speckle tracking imaging
ADO I: Amplatzer duct occluder I
AR: Aortic regurgitation
BMI: Body mass index
BSA: Body surface area
CS: Circumferential strain
CXR: Chest X-ray
ECG: Electrocardiography
EF: Ejection fraction
LAO: Left anterior oblique
LV: Left ventricle
LVEDD: Left ventricle end-diastolic dimension
LVEDV: Left ventricle end-diastolic volume
LVEF: Left ventricle ejection fraction
LVESD: Left ventricle end-systolic dimension
LVESV: Left ventricle end-systolic volume
MR: Mitral regurgitation
RS: Radial strain
RV: Right ventricle
TEE: Transesophageal echocardiography
TR: Tricuspid regurgitation
TTE: Transthoracic echocardiography
VSD: Ventricular septal defect
